# Preparation and Characterization of Electrospun Polysaccharide FucoPol-Based Nanofiber Systems

**DOI:** 10.3390/nano12030498

**Published:** 2022-01-31

**Authors:** Yuliana Vázquez-González, Cristina Prieto, Milan Stojanovic, Cristiana A. V. Torres, Filomena Freitas, Juan Arturo Ragazzo-Sánchez, Montserrat Calderón-Santoyo, Jose M. Lagaron

**Affiliations:** 1 Novel Materials and Nanotechnology Group, Institute of Agrochemistry and Food Technology (IATA), Spanish Council for Scientific Research (CSIC), Calle Catedrático Agustín Escardino Benlloch 7, 46980 Paterna, Spain; yuliana.vg04@gmail.com (Y.V.-G.); milan87stojanovic@gmail.com (M.S.); 2 Laboratorio Integral de Investigación de Alimentos, Tecnológico Nacional de México-Instituto Tecnológico de Tepic, Av. Tecnológico #2595, Tepic 63175, Mexico; arturoragazzo@hotmail.com; 3 UCIBIO-Applied Molecular Biosciences Unit, Department of Chemistry, School of Science and Technology, NOVA University Lisbon, 2819-516 Caparica, Portugal; c.torres@fct.unl.pt (C.A.V.T.); a4406@fct.unl.pt (F.F.); 4 Associate Laboratory i4HB—Institute for Health and Bioeconomy, School of Science and Technology, NOVA University Lisbon, 2819-516 Caparica, Portugal

**Keywords:** FucoPol, exopolysaccharide, electrospinning process, nanofibers, characterization

## Abstract

The electrospinnability of FucoPol, a bacterial exopolysaccharide, is presented for the first time, evaluated alone and in combination with other polymers, such as polyethylene oxide (PEO) and pullulan. The obtained fibers were characterized in terms of their morphological, structural and thermal properties. Pure FucoPol fibers could not be obtained due to FucoPol’s low water solubility and a lack of molecular entanglements. Nanofibers were obtained via blending with PEO and pullulan. FucoPol:PEO (1:3 *w/w*) showed fibers with well-defined cylindrical structure, since the higher molecular weight of PEO helps the continuity of the erupted jet towards the collector, forming stable fibers. WAXS, DSC and TGA showed that FucoPol is an amorphous biopolymer, stable until 220 °C, whereas FucoPol-PEO fibers were stable until 140 °C, and FucoPol:pullulan fibers were stable until 130 °C. Interestingly, blended components influenced one another in intermolecular order, since new peaks associated to intermolecular hierarchical assemblies were seen by WAXS. These results make FucoPol-based systems viable candidates for production of nanofibers for packaging, agriculture, biomedicine, pharmacy and cosmetic applications.

## 1. Introduction

Exopolysaccharides (EPS) are water-soluble, non-toxic, high-molecular-weight polymeric carbohydrate structures composed monosaccharide units joined together by glycosidic bonds [1]. EPS can be homopolysaccharides when composed of a single type of monosaccharide, for example, pullulan; or heteropolysaccharides if they are composed of two or more sugars with different ratios, such as FucoPol [2].

FucoPol is an EPS produced by *Enterobacter* A47, using glycerol as the sole carbon source [3]. It is a high-molecular-weight (4.19 × 10^6^−5.80 × 10^6^) heteropolysaccharide composed of sugar residues [fucose (32–36 mol %), galactose (25–26 mol %), glucose (28–34 mol %) and glucuronic acid (9–10 mol %)], as well as acyl groups [pyruvate (13–14 wt.%), succinate (3 wt.%) and acetate (3–5 wt.%)] [4]. It has an anionic character, with interesting functional properties, including emulsion, film-forming [5], thickening, and flocculating capacity; biological activity and adhesive and photoprotective properties [6,7]. All these properties make FucoPol an interesting candidate for food packaging, agriculture, biomedicine, pharmacy, food and cosmetics applications. Hence, up to now, several research works have studied the potential of FucoPol. For instance, Lourenço et al. evaluated the ability of FucoPol to be used as encapsulation matrix of some bioactive compounds by spray drying [8]. Concordio-Reis et al. [9] studied the stabilization properties of FucoPol in the production of a FucoPol/AgNP biocomposite for wound-healing applications. Additionally, Ferreira et al. [10,11,12,13] studied the development of FucoPol films by casting. These films have shown to be transparent and hydrophilic, with high permeability to water vapor, but presented good barrier properties against oxygen and carbon dioxide.

Electrohydrodynamic processes are commonly used for the formation of micro, submicro and nanostructures. This technology utilizes electrical forces to produce ultrathin fiber-based morphologies from different polymers (natural and synthetic) with diameters ranging from 2 nm to several micrometers. In the electrospinning process, a polymer solution in a capillary tube is subjected to an electric field [14,15]. When the applied electric field reaches a critical value, the repulsive electrical forces overcome the surface tension forces. Eventually, a charged jet of the solution is ejected from the tip of the Taylor cone, and an unstable and rapid whipping jet occurs in the space between the capillary tip and the collector, which leads to evaporation of the solvent at room temperature [14,16,17]. The produced micro and nanostructures may offer many functional advantages, such as superior mechanical properties, flexibility, large surface-to-mass ratio, tailored morphology, tunable porosity and the capability of encapsulation and subsequent release of active and bioactive compounds [18,19,20,21,22,23]. In addition, mechanical properties, thermal stability, electrical conductivity, photocatalytic activity and bioactivity can be tuned by controlling the diameter [23]. These unique characteristics make electrospun micro and nanostructures attractive for novel applications in drug delivery, agriculture, food packaging, biomedical engineering, air filtration, energy production and storage, environmental protection and improvement, photonic and electronic devices [23,24].

To the best of our knowledge, the formation of non-woven FucoPol-based structures by electrospinning has not been reported yet. For this reason, the aim of this research was to study, for the first time, the possibility of producing FucoPol fibers via electrospinning, since the combination of characteristics of the electrospun fibers together with the interesting functional properties of FucoPol could be of great interest for potential applications in food packaging, agriculture, biomedicine, pharmaceutics, food and cosmetics, among others. The electrospinnability of FucoPol was characterized alone and in combination with well-known electrospinnable polymers, such as polyethylene oxide (PEO) and pullulan. The obtained fibers were characterized in terms of their morphological, structural and thermal properties.

## 2. Materials and Methods

### 2.1. Materials

FucoPol (Mw ~ 4.4 × 10^6^ Da) was provided by the Biochemical Engineering Group of Nova School of Sciences and Technology (Caparica, Portugal). FucoPol was produced and purified using the same procedures described by Ferreira et al. and Alves et al. [10,25]. The obtained FucoPol was characterized in terms of its chemical composition, as described by Alves et al. [25]. This heteropolysaccharide was composed of neutral sugars: fucose, galactose and glucose. The relative proportion of sugar monomers was 38% glucose, 25% fucose and 32% galactose. Regarding the content of acyl groups, it reached 12% of the polymer mass, with acetyl, pyruvil and succinyl groups in greatest abundance. The obtained sample also contained non-sugar contaminants, such as proteins and inorganic residues. The protein content was 12 wt.%, and the total content of inorganic residues, determined by pyrolysis at 550 °C, was 32.5%. Regarding the purified polymer’s average molecular weight and polydispersity index, they were determined by size-exclusion chromatography combined with multiple-angle laser light scattering (SEC-MALLS), as described in the work of Hilliou et al. [26]. The average molecular weight was around 1.1 × 10^7^ ± 2.3 × 10^5^ g/mol, with an average polydispersity index of 1.85 ± 0.14.

Pullulan (Mw ~ 2 × 10^5^ Da) was purchased from Hayashibara Co., Ltd. (Tokyo, Japan), and polyethylene oxide (PEO) (Mw ~ 100,000 Da), 2,2,2-trifluoroethanol and Span 20 were purchased from Sigma Aldrich (St. Louis, MO, USA). Absolute ethanol (EPR. Ph.Eur.) was obtained from Labkem (Vilassar de Dalt, Spain). Distillated water was used throughout the study.

### 2.2. Preparation and Characterization of Polymeric Solutions

Solutions of FucoPol were prepared with a concentration of 0.015 g/mL in distilled water, as well as in a mixture of ethanol:water (30:70 *v/v*) at the same concentration. Solubility tests were conducted in absolute ethanol and 2,2,2-trifluoroethanol; however, FucoPol was not soluble in these solvents (results not shown), and consequently, these formulations were not tested in electrospinning. After that, in order to improve the formation of fibers, blends with two well-known electrospinnable polymers were also studied, i.e., PEO and pullulan. Different ratios were studied to find the minimum amount of polymer of high molecular weight required to generate the molecular entanglements necessary to obtain the sought fibers. In order to reduce surface tension and to make the process more stable to obtain suitable fibers, ethanol–water solution (30:70 *v/v*) was used for the solutions with PEO and pullulan. The concentration of FucoPol in the solutions was maintained at 0.015 g/mL, and different ratios were explored, as shown in Table 1. Span 20 was used as a surfactant at a concentration of 3% (*w/w*). All formulations were proper solutions without solid residues.

Characterization of the solutions with the best electrospinning processing was made in terms of viscosity, conductivity and surface tension. Viscosity was measured using a Visco Basic Plus L rotational viscometer (Fungilab S.A., Sant Feliu de Llobregat, Spain). Surface tension was determined in an Easy Dyne K20 tensiometer (Krüss GmbH, Hamburg, Germany) following the Wilhelmy plate method. Conductivity was measured using a conductivity meter (HI-4521 portable meter, Hanna Instruments, Gothenburg, Sweden). Measurements were taken in triplicate at room temperature.

### 2.3. Electrospinning Process

The electrospinning process was performed in a high-throughput Fluidnatek^®^ LE-500 from Bioinicia S.L. (Valencia, Spain). The solutions of FucoPol in water and FucoPol in ethanol:water (30:70 *v/v*) were processed using different processing conditions in order to obtain fibers. The studied range of operation parameters was: flow rate between 250 and 500 µL/h, work distance between 15 and 30 cm, and voltage between 16 and 23 kV. Temperature and relative humidity (RH) were controlled at 35 °C and 28%, respectively.

### 2.4. Characterization of Electrospinning Fibers 

#### 2.4.1. Scanning Electron Microscopy (SEM) 

The morphology of the obtained fibers was determined using a Hitachi-S-4800 FE-SEM scanning electron microscope (Hitachi High-Technologies Corporation, Tokyo, Japan) with an electron-beam acceleration of 10 kV. Approximately 1.5 mg of sample was placed using double-sided tape on the sample holder and coated with a gold-palladium layer. Fiber sizes were analyzed using Image J Launcher v1.41 software (National Institutes of Health, Bethesda, MD, USA). The presented data are based on measurements from a minimum of 100 fibers.

#### 2.4.2. Attenuated Total Reflectance Fourier Transform Infrared Spectroscopy (ATR-FTIR)

To evaluate possible interactions between the different polymers that integrated the fibers, ATR-FTIR (Bruker FTIR Tensor 37 equipment, Rheinstetten, Germany) was used. The samples were placed on top of the diamond crystal, and appropriate contact was assured by using the Golden Gate low-temperature ATR sampling accessory (Specac Ltd., Orpington, UK). All spectra were obtained within the wavenumber range of 4000–600 cm^−1^ by averaging 10 scans at 4 cm^−1^ resolution. Analysis of spectral data was carried out using Origin Pro, Version 2019 (OriginLab Corporation, Northampon, MA, USA).

#### 2.4.3. Differential Scanning Calorimetry (DSC)

Thermal transitions were studied by differential scanning calorimetry (DSC) on a DSC-8000 analyzer equipped with the Intracooler 2 cooling accessory from PerkinElmer, Inc. (Waltham, MA, USA). Three sweeps were done: a first heating step from 30 °C to 250 °C, followed by a cooling step from 250 to −25 °C and completed by a second heating from −25 to 300 °C. The heating and cooling rates were both set at 10 °C/min, and the sample weight was around 3 mg. An empty aluminum pan was used as reference, whereas calibration was performed using an indium sample. The values of melting point (Tm) and enthalpy of melting (ΔHm) were obtained from the heating scan, while the crystallization temperature from melt (Tc) and enthalpy of crystallization (ΔHc) was determined from the cooling scan.

#### 2.4.4. Wide-Angle X-ray Scattering (WAXS)

The crystallinity of the fibers and pure polymers were assessed by WAXS using a Bruker AXS D4 Endeavor diffractometer (Bruker, Ettlingen, Germany), similarly to a previous work [27]. Radial scans of intensity versus scattering angle (2θ) were recorded at room temperature in the range of 2 to 40° (2θ) (step size = 0.02° (2θ), scanning rate = 4 s/step) with filtered CuKα radiation (λ = 1.54 Å), an operating voltage of 40 kV and a filament current of 40 mA.

#### 2.4.5. Thermogravimetric Analysis (TGA)

Thermogravimetric analysis of all polymers and fibers was done in triplicate using a 550-TA thermogravimetric analyzer (New Castle, DE, USA). The analyses were carried out under the following conditions: 1–5 mg of sample, heating from 25 °C to 600 °C, at a heating rate 10 °C/min under nitrogen flow (50 mL/min).

### 2.5. Statistical Analysis

Data were analyzed by ANOVA with a *p*-value < 0.05. Fisher test was used for the comparison of means. STATISTICA 10 software (Statsoft Inc., Tulsa, OK, USA) was used for statistical analysis.

## 3. Results and Discussion

The aim of this work was to assess the electrospinnability of FucoPol and its blends with PEO and pullulan.

### 3.1. Physicochemical Characterization of Solutions

A fundamental step before electrospinning is to physicochemically characterize the solutions. These parameters govern the behavior of the solution throughout the process. Solutions of FucoPol in different solvents and FucoPol with complementary polymers were characterized in terms of viscosity, surface tension and conductivity. Table 2 shows the characterization of the solutions of pure FucoPol, as well as the solutions of FucoPol:pullulan (2:1 ratio, *w/w*) and FucoPol:PEO (1:3 ratio, *w/w*). Viscosity is an important parameter in the electrospinning process in terms of good fiber formation, since below a critical value, the polymeric fibers break up the jet and form droplets on their way to the collector. However, a high viscosity makes it difficult to pass through the syringe needle of the equipment and stabilize the electrospinning process [28,29]. FucoPol water solutions showed a viscosity of 24.8 ± 0.3 Pa·s. This value is low in comparison with values reported in the literature. Araújo et al. [6], reported a value of 43 Pa·s, but the difference could be due to the EPS extraction process, since some authors decrease the viscosity of the bacterial broth, either through dilutions with distilled water or by adjusting pH with H_2_SO_4_ to increase the production of EPS [9,30].

The increased viscosity of FucoPol:pullulan solution, 38.9 ± 0.1 Pa·s, is attributed to the addition of pullulan and the higher percentage of solids in the final solution (2.25% in comparison with 1.50% for FucoPol). In addition, ethanol increased the viscosity of the solutions of FucoPol with pullulan and FucoPol with PEO in comparison with the same solutions prepared with water (results not shown). The solution of FucoPol with PEO showed a viscosity of 98.1 ± 0.5 Pa·s. This value was statistically significant in comparison with the previous solutions. This behavior was mainly attributed to the high molecular weight of PEO.

FucoPol had a surface tension of 64.20 ± 0.40 mN/m. This value was high and does not ensure stability during the electrohydrodynamic process [31]. A decrease in surface tension occurred when a mixture of water and ethanol (70:30, *v/v*) was used instead of pure water, as shown in Table 2. In other words, the solutions with pullulan and PEO showed values of 41.00 ± 0.40 mN/m and 43.90 ± 0.10 mN/m, respectively, since the surface tension of each solution is dependent on the polymer and the solvent used and on the presence of Span 20 [15].

In the same way, conductivity decreased when ethanol was added to the solutions with pullulan and PEO. Highly conductive solutions, such as FucoPol, are extremely unstable in the presence of strong electric fields, and led to a high bending instability [32].

### 3.2. Production of Nanofibers by Electrospinning 

**Figure 1:**
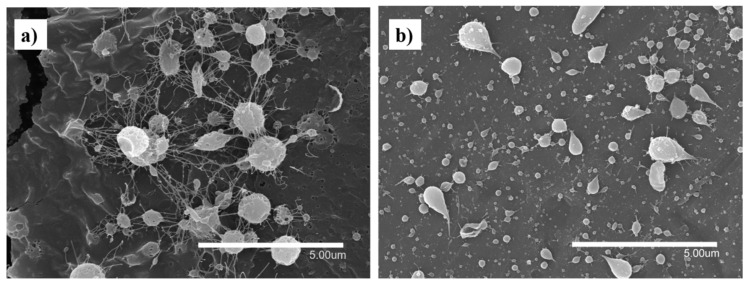
Fibers and capsules of FucoPol in water. (**a**) (optimal conditions: 500 µL/h, 25 cm between collector and needle and 21 kV) and ethanol:water, (**b**) (optimal conditions: 500 µL/h, 15 cm between collector and needle and 15.5 kV in the injector and −3.6 kV in the collector).

**Figure 2:**
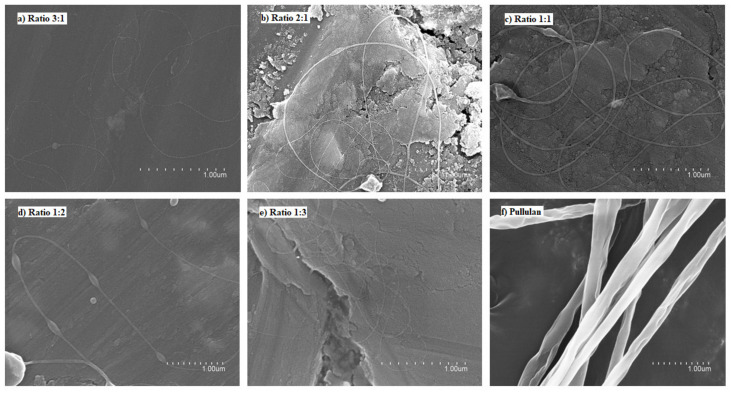
Scanning electron microscopy (SEM) images of the fibers obtained with the FucoPol:pullulan solutions. (**a**) 3:1 ratio, (**b**) 2:1 ratio, (**c**) 1:1 ratio, (**d**) 1:2 ratio, (**e**) 1:3 ratio, (**f**) 0:1 ratio (100% pullulan).

In spite of using different processing conditions, it was not possible to obtain fibers using pure FucoPol in water (Figure 1a) and ethanol:water (30:70 *v/v*) (Figure 1b) due to the low solubility of the biopolymer in water [10,11,12,13], which produces a low solid concentration in the solution, as well as the lack of polymer chain entanglements [33]. For this reason, different ratios with other polymers and process conditions were evaluated. Figure 2 shows the micrographs obtained by SEM of the different solutions of FucoPol:pullulan. Figure 2a–e shows fibers with cylindrical structure but with the presence of some beads, which are more visible at a ratio of 1:2 (Figure 2d). Sinuous fibers occur by warping the jet due to the impact with the collector plate, which is also closely related to the viscosity of the solution and the rigidity of the jets [34]. Bead formation occurs mainly due to the high surface tension and viscoelastic properties of the solution, as well as the operating parameters used [19], affecting the homogeneity of the fibers.

On the other hand, pullulan showed uniform and cylindrical fibers with a rough surface, which could be caused by a combination of viscosity of the solution and the ambient conditions. This type of fiber is characteristic of pullulan due to the composition of the polymer. Pullulan has a single bond of maltotriose units interconnected by glycosidic bonds α (1,4) and α (1,6). This structure provides adhesive properties, as well as the ability to form fibers, compression molds and strong oxygen-impermeable films [2].

The studied ratios, shown in Figure 2, did not show significant differences, since the obtained fibers showed similar morphology and nanometric size. Although the different ratios of FucoPol:pullulan showed beads, these fibers were better than the fibers obtained with only FucoPol. FucoPol:pullulan nanofibers with a mass ratio of 2:1 (*w/w*) were selected to continue the study. These fibers were obtained at a constant flow rate of 250 µL/h, voltages of 23 kV in the injector and −2 kV in the collector and distance between needle and collector of 28 cm.

**Figure 3:**
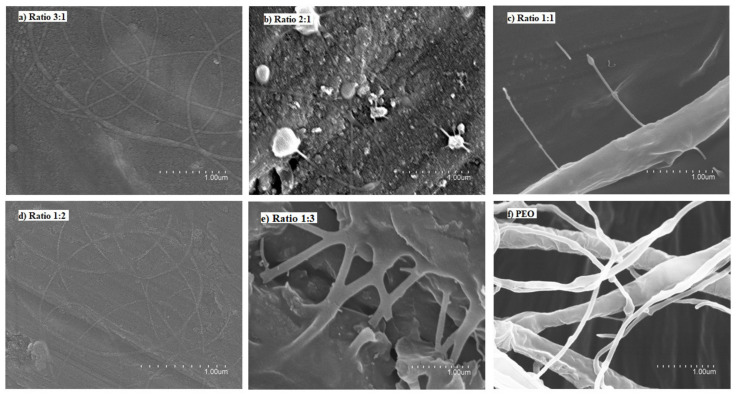
Scanning electron microscopy (SEM) images of the fibers obtained with the FucoPol:PEO solutions. (**a**) 3:1 ratio, (**b**) 2:1 ratio, (**c**) 1:1 ratio, (**d**) 1:2 ratio, (**e**) 1:3 ratio, (**f**) 0:1 ratio (100% PEO).

**Figure 4:**
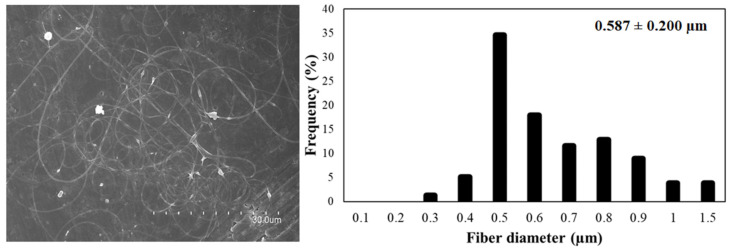
FucoPol:PEO fibers (1:3 ratio, *w/w*) (left) and the average diameter of fibers (right).

Figure 3 shows the micrographs of the different solutions of FucoPol:PEO. Ratios of 3:1 and 1:2 (Figure 3a,d, respectively) showed fibers with a well-defined cylindrical structure and no beads. However, when samples were collected for a long time, fibers and particles were observed, similarly to the fibers obtained with FucoPol:pullulan solutions. Ratios of 2:1 and 1:1 did not show a stable process (Figure 3b and Figure 3c, respectively). Fiber branching observed in Figure 3c,f was due to the high voltage required to electrospin these solutions, which provoked an excess of surface charge on the jet, which was dissipated by branching [35]. However, a ratio of 1:3 led to thick, smooth, continuous and homogeneous fibers due to the higher amount of PEO (Figure 3e and Figure 4). Even if some defects could be detected in Figure 4, they were much less numerous in comparison to the thicker films obtained with the other samples. The solution of FucoPol with PEO (1:3 ratio *w/w*) showed nanofibers with an average diameter of 0.587 ± 0.200 µm. Those fibers were obtained at a constant flow rate of 300 µL/h, with voltages of 22 kV in the injector and −2 kV in the collector and a distance between the needle and the collector of 28 cm. PEO is considered an electrospinnable polymer [29], since it improves the structural stability and the charge-carrying capacity of the solution, which is crucial for fiber formation [36]. This result is in agreement with previous studies that showed that the higher viscosity of the solution increases the diameter of fibers [15].

### 3.3. Thermal Properties 

#### 3.3.1. Thermal Gravimetric Analysis (TGA)

**Figure 5:**
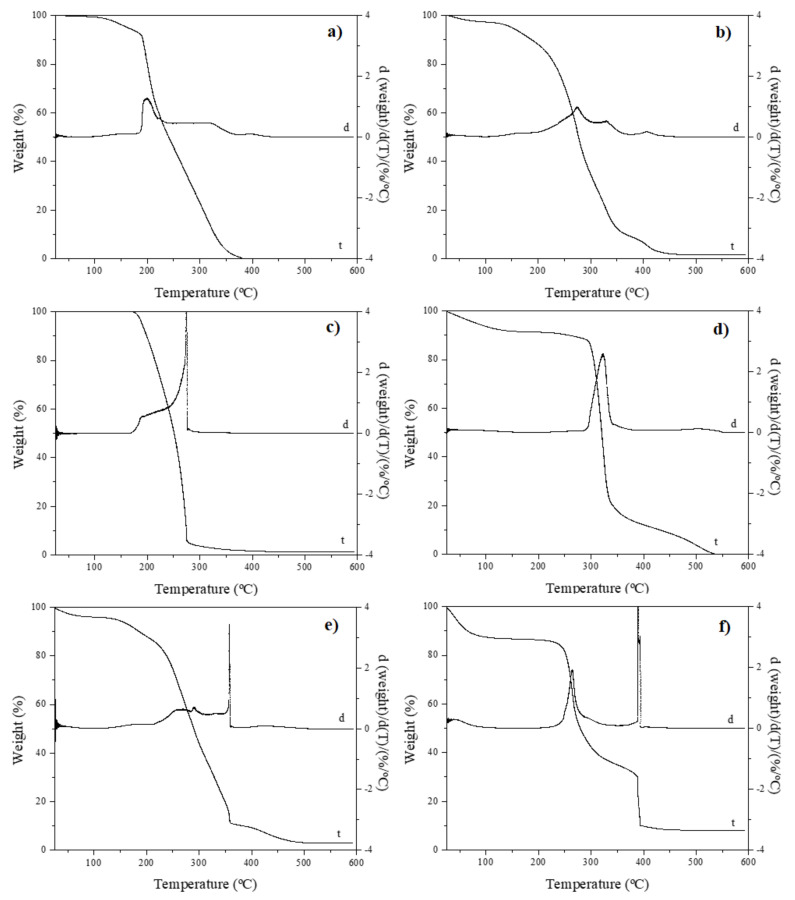
Thermogravimetric analysis of (**a**) FucoPol: PEO fibers, (**b**) FucoPol:pullulan fibers, (**c**) pure PEO, (**d**) pure pullulan, (**e**) FucoPol after electrospinning (mixture of fibers and particles) and (**f**) pure FucoPol. Curve t represents thermogram, and d is the thermogram derivate.

Thermogravimetric curves of FucoPol, FucoPol:pullulan (ratio 2:1, *w/w*) and FucoPol:PEO (ratio 1:3, *w/w*), along with TGA curves of pure polymers, which served as references, are shown in Figure 5. FucoPol showed three weight-loss steps (Figure 5f). The first step occurred between 25 and 109 °C, referring to 13% of weight loss, which could be attributed to water evaporation [8,9]. The second step occurred between 220 and 364 °C, referring to 52% of weight, and the third step occurred between 364 and 410 °C, referring to 23% of weight loss. These two steps were attributed to biopolymer thermal degradation [8,37], specifically to decomposition of the polysaccharides side chains [9]. After these three steps, we observed a gradual weight loss, around 10% of total mass. It is important to note that after the electrospinning process, FucoPol showed only two weight-loss steps (Figure 5e), starting from 25–109 °C, corresponding to 4% of weight loss, attributed to the evaporation of water. The second step started from 140–378 °C, referring to 85% of weight loss, and after this, a gradual weight loss. This behavior is thought to be caused by the reduced size of the fibers and the presence of 3% Span 20. The oily and hydrophobic nature [38] of Span 20 leads to a wide range of decomposition temperatures compared to pure FucoPol.

When it comes to FucoPol:PEO fibers, a similar thermal-degradation curve as for pure PEO is observed (Figure 5c). The thermal decomposition of PEO and FucoPol:PEO fibers consisted of only one weight-loss step. PEO showed 96% of weight loss between 160 and 301 °C, while FucoPol:PEO fibers (Figure 5a) showed a step between 143 and 379 °C with a similar weight loss (97%). The presence of PEO decreased the thermal stability of the fibers in comparison to pure FucoPol nanofibers.

Pullulan showed one important weight-loss step from 267 to 362 °C, corresponding to 74% of total weight loss (Figure 5d). Nevertheless, FucoPol:pullulan fibers showed a comparable thermal behavior to that of FucoPol after electrospinning (Figure 5e), attributed to the largest amount of FucoPol among the FucoPol:pullulan fibers (2:1 *w/w*). Weight loss started from 128 to 369 °C, corresponding to 87% of total weight loss. This study confirms that the obtained nanofibers could be used in multiple applications in which the temperature does not exceed 120 °C. However, this temperature is enough to be subjected to several thermal processes, such as sterilization, as well as to non-thermal technologies, such as electrohydrodynamic processing, which opens a wide range of potential applications, such as pharmaceutics, biomedicine, biosensors, food packaging, agriculture, food or cosmetics, among others.

#### 3.3.2. Differential Scanning Calorimetry (DSC) 

**Figure 6:**
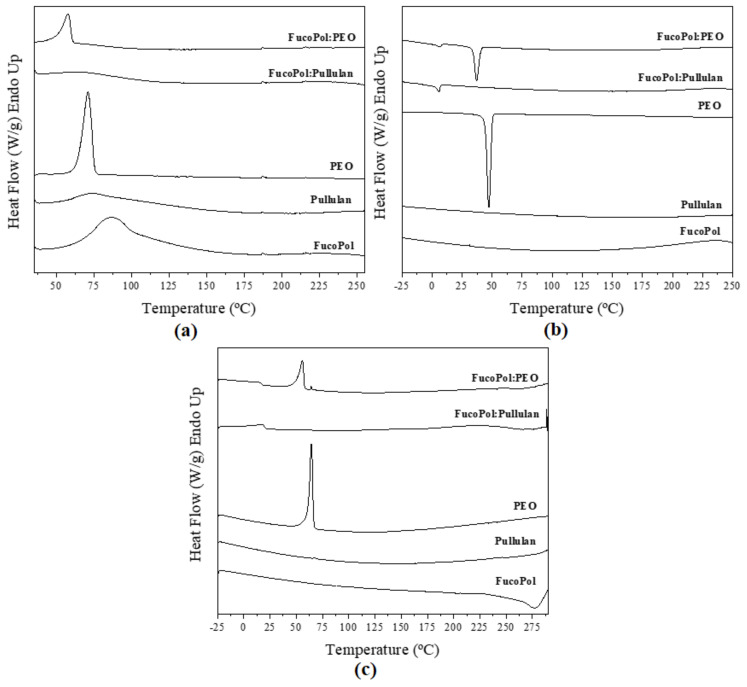
Differential scanning calorimetry curves of electrospun fibers and commercial polymers during (**a**) first heating, (**b**) cooling, and (**c**) second heating.

DSC curves of fibers are illustrated in Figure 6. The DSC curve of pure FucoPol during the first run showed an endothermic peak at 85 °C, ΔH 113 J/g (Figure 6a), which was also observed by Lourenço et al. [8]. This may be attributed to the evaporation of sorbed water. During the second heating run, stopping at a higher temperature than in the first run, an exothermic peak at 279 °C, ΔH −144 J/g (Figure 6c), was observed, which was attributed to polymer degradation. Additionally, Lourenço et al. reported an exothermic peak at around 270 °C [8]. Unambiguous melting or crystallization events were not discerned in the different heating and cooling ramps, suggesting an amorphous nature of the biopolymer. The same behavior was observed by Guerreiro et al. [39]. 

In the same way, pullulan showed a similar first-heating thermogram, with a broad peak between 44 and 166 °C instead of a sharp melting point (Figure 6a). This endotherm is also ascribed to moisture evaporation. The same behavior was observed by Singh et al. [40].

On the other hand, PEO is a semicrystalline polymer [41]. Figure 6a exhibits the melting temperature of PEO at 69 °C, ΔH 151 J/g. Crystallization temperature was 47 °C, ΔH 122 J/g (Figure 6b). Similar results were reported by Balik et al. [42]. The FucoPol:PEO fibers showed similar behavior to that of pure PEO because PEO was the dominant polymer in the solution (1:3 ratio, *w/w*, Table 1). However, melting and crystallization temperatures and enthalpies corrected for PEO content were reduced, i.e., 58 °C, ΔH 76 J/g and 37 °C, ΔH 63 J/g, respectively, as shown in Figure 6a and Figure 6b. This behavior was attributed to FucoPol impairing the crystallization of the PEO polymer in the blend. Guerreiro et al. [39] reported the formation of dispersed ice crystals in the presence of FucoPol when they studied the cryoprotective properties of FucoPol in water solutions. These authors observed that FucoPol significantly reduced the average size of the formed crystals.

Interestingly, during the crystallization and melting runs of the blends, small exothermic and endothermic events were seen at 10.5 and 17.5 °C, respectively, for FucoPol:pullulan and at 8.1 and 15.5 °C, respectively, for FucoPol:PEO, which may be ascribed to higher hierarchical molecular assemblies induced by blending (see WAXS results below). 

### 3.4. Wide-Angle X-ray Scattering (WAXS)

**Figure 7:**
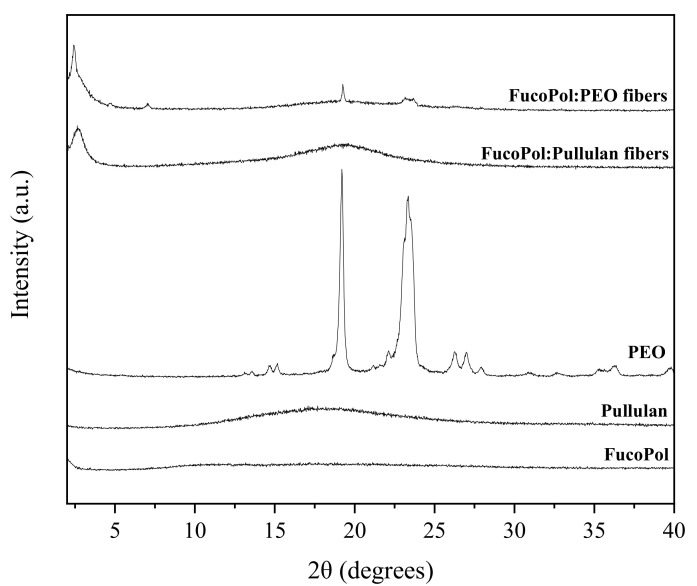
WAXS profiles of fibers of FucoPol with PEO and pullulan, respectively, as well as pure polymers.

WAXS allows for the characterization and quantification of the crystallinity present in a given sample through the diffraction patterns measured at wide angles [43]. Results of this analysis are shown in Figure 7. In this context, pure FucoPol and pullulan showed no sharp peaks in the 2θ range, suggesting again a rather amorphous nature [8,44,45,46,47,48]. On the other hand, pure PEO showed a clear crystalline phase with two intense peaks at 19° and at 23° (Figure 7). These observations are consistent with the results obtained by other authors, who reported two characteristic diffraction peaks at 2θ = 19° and 23.5° for crystalline PEO [49] and weak crystalline peaks at around 15°, 26° and 36° [50,51]. These peaks are attributed to the well reported ordered 7/2 helical structure of PEO, i.e., seven ethylene oxide repeat units with two turns in a fiber period of 1.93 nm [51]. In the FucoPol:PEO fibers, the crystalline patterns of PEO are weaker than in the pure polymer but can be clearly discerned. In addition, some new peaks were detected in both blends at angles below 10°. In the case of FucoPol:PEO fibers, they exhibited a new tiny peak at 7°, which suggests that PEO could affect the FucoPol biopolymer hierarchical assembly. Additionally, both blends show a stronger peak at around 3°. Peaks at these low angles have been attributed to helical secondary structures, as well as other higher hierarchical assemblies by other authors [52,53,54].

### 3.5. Attenuated Total Reflectance–Fourier Transform Infrared Spectroscopy (ATR-FTIR) 

**Figure 8:**
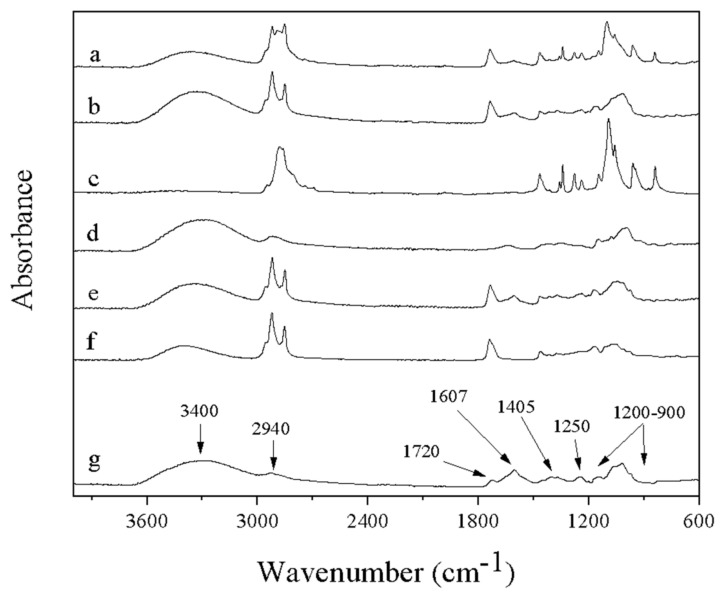
FTIR spectra of (**a**) FucoPol:PEO fibers, (**b**) FucoPol:pullulan fibers, (**c**) pure PEO, (**d**) pure pullulan, (**e**) FucoPol after electrospinning (mixture of fibers and particles), (**f**) Span 20 and (**g**) pure FucoPol.

The ATR-FTIR spectra of the fibers that contain FucoPol are presented in Figure 8, along with the spectra of complementary polymers pullulan and PEO for comparison. The broad intense band around 3400 cm^−1^ is common for all samples containing polysaccharides (Figure 8a,b,d,e,g). This band represents O–H stretching of hydroxyls and bound water [55]. This band overlaps in part with the C–H stretching peak of CH_2_ groups appearing at 2940 cm^−1^ in the case of FucoPol and pullulan, as shown in Figure 8g and Figure 8d, respectively. FucoPol bands at 1200 and 900 cm^−1^ represent skeletal C–O and C–C vibration bands of glycosidic bonds and pyranoid rings [55]. The band at 1720 cm^−1^ is attributed to the C=O stretching of carbonyls in acyl groups. The band at 1250 cm^−1^ may also be attributed to the C–O–C vibration of acyls and was explored by Freitas et al. [7]. In the same way, the strong bands at 1607 and 1405 cm^−1^, can be attributed to the asymmetric and symmetric stretchings, respectively, of carboxylates [55]. All peaks shown by FucoPol (Figure 8g) in this study match those reported by Freitas et al. [7] during the characterization of a fucose-containing exopolysaccharide produced by the newly isolated *Enterobacter* strain A47 DSM 23139.

The fibers of FucoPol:PEO and FucoPol:pullulan showed characteristic bands corresponding to the same bands of pure polymers PEO and pullulan, respectively (Figure 8c,d). In addition, the presence of the Span 20 was also observed, since new bands appeared in the spectrum of fibers at ca. 2920 and 2850 cm^−1^, attributed to CH_2_ and CH_3_ scissoring [56] of the lipids that form this surfactant, even in FucoPol after electrospinning. The absence of changes in the characteristic bands in the electrospun nanofibers with respect to the pure components proves the absence of chemical reactions and degradation during the applied treatment.

## 4. Conclusions

Electrospun FucoPol nanofibers were not able to be produced from water or alcohol solutions—only by blending with other polymers, such as PEO and pullulan. FucoPol was found to be an amorphous biopolymer stable until 220 °C, but it was not possible to obtain homogeneous nanofibers due to the low water solubility and lack of molecular entanglements. The blend of FucoPol:PEO showed non-agglomerated and homogeneous nanofibers with cylindrical morphology, as well as reduced crystallinity in comparison with pure PEO fibers when normalized for content, and was stable until 140 °C. The blend of FucoPol:pullulan showed an amorphous nature and was stable until 130 °C. Interestingly, the blending components influenced one another in the intermolecular order, since new peaks ascribed to intermolecular hierarchical assemblies were seen by WAXS for FucoPol. The well-known functional properties of FucoPol, together with the possibility to form stable electrospun fibers with controllable morphology and porosity, as well as the interesting structural and thermal properties shown in previous results, opens a new research line concerning the potential applications of FucoPol electrospun fibers in the food packaging, agricultural, biomedical, pharmaceutical, cosmetic and food industries, among others.

## Figures and Tables

**Table 1:** Polymer ratio employed for the preparation of the nanofibers via electrospinning. All solutions contained 3% of Span 20 as a surfactant.

FucoPol/PEO (*w/w*)	FucoPol/Pullulan (*w/w*)
3:1	3:1
2:1	2:1
1:0	1:0
0:1	0:1
1:2	1:2
1:3	1:3

**Table 2:** Physicochemical properties of different dissolutions: FucoPol in water, FucoPol with pullulan (2:1 ratio, *w/w*) and FucoPol with PEO (1:3 ratio, *w/w*) in ethanol:water (30:70 *v/v*).

Sample	Viscosity * (Pa·s)	Surface tension (mN/m)	Conductivity (mS/cm)
FucoPol in water	24.8 ± 0.3 ^c^	64.20 ± 0.40 ^a^	1.55 ± 0.00 ^a^
FucoPol + Pullulan, ratio 2:1	38.9 ± 0.1 ^b^	41.00 ± 0.40 ^c^	0.53 ± 0.00 ^b^
FucoPol + PEO, ratio 1:3	98.1 ± 0.5 ^a^	43.90 ± 0.10 ^b^	0.50 ± 0.01 ^c^

Each column represents the mean ± standard deviation of three independent replicas. Different letters (a–c) in each column indicate a significant difference (*p* < 0.05). * All samples were Newtonian fluids.

## Data Availability

No application.
